# A Novel Screening System for Claudin Binder Using Baculoviral Display

**DOI:** 10.1371/journal.pone.0016611

**Published:** 2011-02-14

**Authors:** Hideki Kakutani, Azusa Takahashi, Masuo Kondoh, Yumiko Saito, Toshiaki Yamaura, Toshiko Sakihama, Takao Hamakubo, Kiyohito Yagi

**Affiliations:** 1 Laboratory of Bio-Functional Molecular Chemistry, Graduate School of Pharmaceutical Sciences, Osaka University, Suita, Osaka, Japan; 2 Department of Molecular Biology and Medicine, Research Center for Advanced Science and Technology, The University of Tokyo, Meguro, Tokyo, Japan; University of South Florida College of Medicine, United States of America

## Abstract

Recent progress in cell biology has provided new insight into the claudin (CL) family of integral membrane proteins, which contains more than 20 members, as a target for pharmaceutical therapy. Few ligands for CL have been identified because it is difficult to prepare CL in an intact form. In the present study, we developed a method to screen for CL binders by using the budded baculovirus (BV) display system. CL4-displaying BV interacted with a CL4 binder, the C-terminal fragment of *Clostridium perfringens* enterotoxin (C-CPE), but it did not interact with C-CPE that was mutated in its CL4-binding region. C-CPE did not interact with BV and CL1-displaying BV. We used CL4-displaying BV to select CL4-binding phage in a mixture of a scFv-phage and C-CPE-phage. The percentage of C-CPE-phage in the phage mixture increased from 16.7% before selection to 92% after selection, indicating that CL-displaying BV may be useful for the selection of CL binders. We prepared a C-CPE phage library by mutating the functional amino acids. We screened the library for CL4 binders by affinity to CL4-displaying BV, and we found that the novel CL4 binders modulated the tight-junction barrier. These findings indicate that the CL-displaying BV system may be a promising method to produce a novel CL binder and modulator.

## Introduction

Tight junctions (TJ) are intercellular adhesion complexes in epithelial and endothelial cells; TJs are located in the most apical part of the complexes [1]. TJs have a barrier function and a fence function [2]–[4]. TJs contribute to epithelial and endothelial barrier functions by restricting the diffusion of solutes through the paracellular pathway. TJs maintain cellular polarity by preventing the free movement of membrane proteins between the apical and basal membranes [5]. Loss of cell-cell adhesion and cellular polarity commonly occurs in the early stages of cancer [6]. Modulation of the TJ barrier function can be a method to enhance drug absorption, and TJ components exposed on the surface of cancer cells can be a target for cancer therapy.

Biochemical analyses of TJs have identified TJ components, such as occludin, claudins (CLs) and junction adhesion molecule [7]. The CL family contains more than 20 integral tetra-transmembrane proteins that play pivotal roles in the TJ barrier and fence functions. CL1-deficient mice lack the epidermal barrier, while CL5-deficient mice lack the blood-brain barrier [8], [9], indicating that the regulation of the TJ barrier by modulation of CLs may be a promising method for drug delivery. *Clostridium perfringens* enterotoxin (CPE) causes food poisoning in humans [10]. An interaction between the C-terminal domain of CPE (C-CPE) with CL4 deregulates the TJ barrier [11], [12]. We previously found that C-CPE enhances jejunal absorption through its interaction with CL4, indicating that a CL binder is a potent drug-delivery system [13].

The majority of lethal cancers are derived from epithelial tissues [14]. Malignant tumor cells frequently exhibit abnormal TJ function, followed by the deregulation of cellular polarity and intercellular contact, which is commonly observed in both advanced tumors and the early stages of carcinogenesis [6]. Some CLs are overexpressed in various types of cancers. For example, CL3 and CL4 are overexpressed in breast, prostate, ovarian, pancreatic and gastric cancers. CL1, CL7, CL10 and CL16 are overexpressed in colon, gastric, thyroid and ovarian cancers, respectively [15], [16]. These findings indicate that the CLs may be a target molecule for cancer therapy. A receptor for CPE is CL4 [11], [12]. CPE has anti-tumor activity against human pancreatic and ovarian cancers without side effects [17], [18]. The CLs binders will be useful for cancer-targeting therapy.

As above, recent investigations of CLs provide new insight into their use as pharmaceutical agents; for example, a CL binder may be used in drug delivery and anti-tumor therapy. Selection of a CL binder by using a recombinant CL protein is a putative method to prepare a CL binder. However, CLs are four-transmembrane proteins with high hydrophobicity; there has been little success in the preparation of intact CL protein. Recently, a novel type of protein expression system that uses baculovirus has been developed. Membrane proteins are displayed on the budded baculovirus (BV) in their active form [19]–[21], indicating that the BV system may be useful for the preparation of a CL binder. In the present study, we investigated whether a CL binder was screened by using a CL-displaying BV.

## Results

### Preparation of CL4-displaying BV

**Figure 1:**
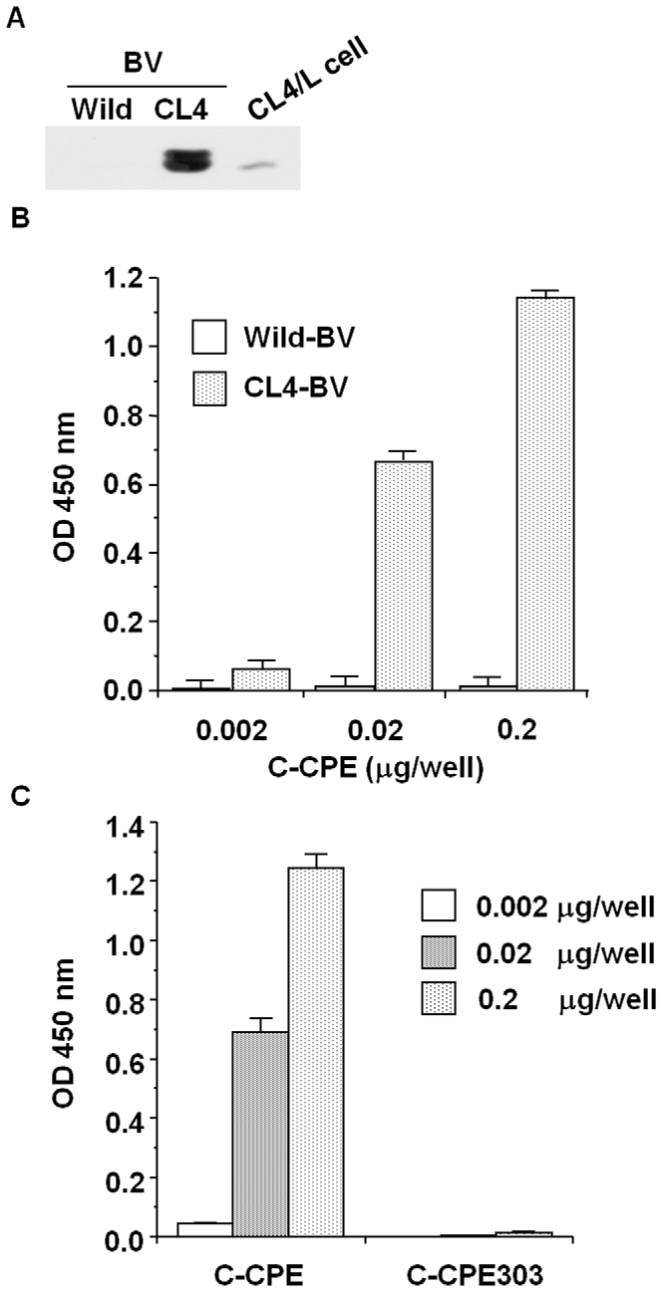
Preparation of CL4-displaying BV. A) Immunoblot analysis. Wild-BV and CL4-BV (0.1 µg/lane) were subjected to SDS-PAGE, followed by immunoblot analysis with anti-CL4 antibody. The lysate of CL4-expressing L (CL4/L) cells was used as a positive control. B, C) Interaction of a CL4 binder with CL4-BV. Immunotubes were coated with the wild-BV or CL4-BV, and C-CPE (B) or mutated C-CPE (C) was added to the BV-coated immunotubes at the indicated concentration. C-CPE bound to the BV-coated tubes was detected by ELISA with an anti-his-tag antibody.

C-CPE is the only known CL binder and modulator [12], [13], [22]. C-CPE has affinity to CL4 in a nanomolar range [23]. We chose C-CPE and CL4 as models of the CL binder and CL, respectively. Several reports indicate that membrane proteins expressed on the surface of BV are in an intact form [19]–[21]. To check the expression of CL4 on the BV, we performed immunoblot analysis of the lysate of CL4-BV against CL4. As shown in Fig. 1A, CL4 was detected in the virus lysates. To determine if the CL4 expressed on the virus has an intact form, we performed enzyme-linked immunosorbent assay (ELISA) with CL4-BV-coated immunoplates. C-CPE binds to the extracellular loop domain of CL4 [23]. After the addition of C-CPE to the CL4-BV-coated plate, the C-CPE bound to the CL4-BV-coated plate was detected by anti-his-tag antibody, followed by incubation with horseradish peroxidase-labeled antibody. C-CPE was dose-dependently bound to CL4-BV, whereas C-CPE did not interact with wild-BV (Fig. 1B). Deletion of the CL4-binding region (C-CPE303) attenuated the interaction of C-CPE with CL4-BV (Fig. 1C). Together, these results indicate that the CL4 displayed on BV may have an intact extracellular loop region.

### Selection of C-CPE-phage by using CL4-BV

**Figure 2:**
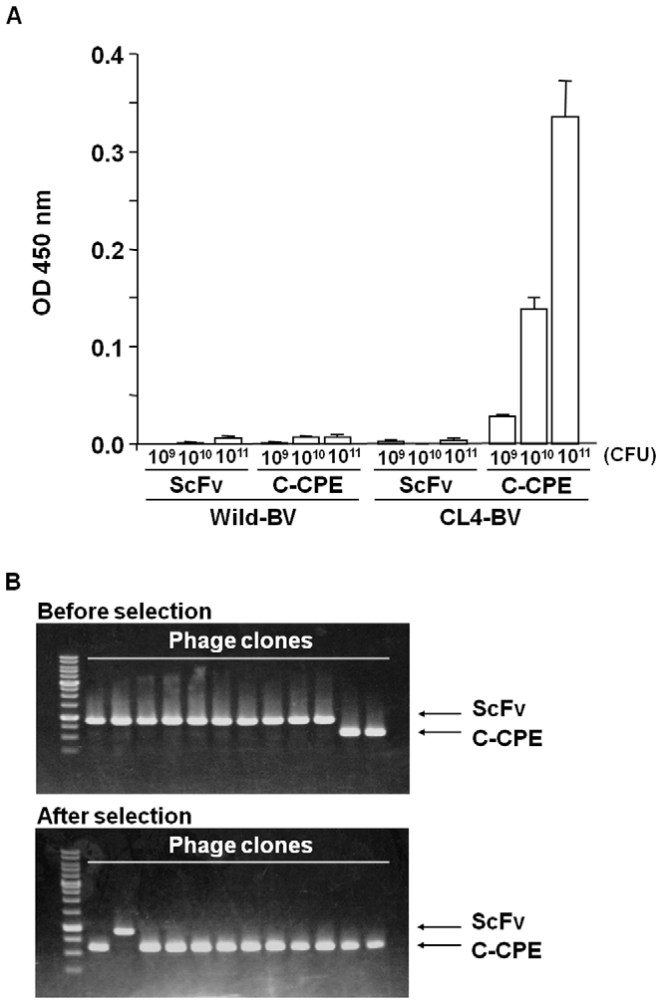
Selection of C-CPE-displaying phage by using the CL4-BV system. A) Interaction of C-CPE-displaying phage with CL4-BV. Wild-BV or CL4-BV was coated on an immunoplate, and then scFv-displaying phage or C-CPE-displaying phage was added to the BV-coated immunoplate at the indicated concentrations. The BV-bound phages were detected by ELISA with anti-M13 antibody as described in Materials and methods. Data are representative of two independent experiments. Data are means ± SD (n = 3). B) Enrichment of C-CPE-displaying phage by the BV system. A mixture of scFv-phage and C-CPE-phage (mixing ratio of scFv-phage to C-CPE-phage = 2∶10) was incubated with a CL4-BV-coated immunotube, and the bound phages were recovered. Each phage clone was identified by PCR amplification, followed by agarose gel electrophoresis. Upper and lower pictures are before and after the selection, respectively. The putative sizes of the PCR products are 856 and 523 bp in scFv and C-CPE, respectively. The data are representative of two independent experiments.

We next examined the interaction between C-CPE-phage and CL4-BV. As shown in Fig. 2A, C-CPE-phage bound to CL4-BV but not to wild-BV, and a scFv-phage did not bind to CL4-BV. To determine if CL-BV can be used to select CL binders, we prepared a mixture of C-CPE-phage and scFv-phage at a ratio of 2∶10 and used CL4-BV to select CL4-binding phage in the mixtures. The amount of C-CPE-phage was increased to 11 of 12 clones in the mixture (Fig. 2B), indicating that CL-BV may be useful in the preparation of CL binders.

We previously found that each substitution of S304, S305, S307, N309, S313 and K318 with alanine increased the binding of C-CPE to CL4 [24]. Here, we prepared a phage library for C-CPE by randomly changing the functional 6 amino acids to any of the 20 amino acids. To confirm the diversity of the library, we checked the sequences of 17 randomly isolated clones. Each of the 17 clones had a different sequence, indicating that the library has a diverse population of C-CPE mutants (Table 1).

**Table 1:** C-CPE phage library.

	304	305	307	309	313	318
C-CPE	S	S	S	N	S	K
Clone 1	V	T	C	V	N	K
2	C	P	A	H	L	T
3	A	G	G	V	P	P
4	R	G	H	L	E	H
5	A	A	P	S	R	Q
6	P	A	P	D	P	A
7	C	T	T	T	N	K
8	H	P	S	P	G	H
9	R	G	G	R	N	R
10	A	P	S	T	Q	P
11	V	L	G	N	M	R
12	P	P	A	T	F	R
13	G	D	C	S	N	L
14	F	R	V	F	R	N
15	S	Q	Q	W	T	T
16	S	R	L	E	W	Q
17	K	R	E	R	Q	S

Phage clones were randomly picked up from the C-CPE phage library, and the amino acids sequences of C-CPE mutant were analyzed.

**Figure 3:**
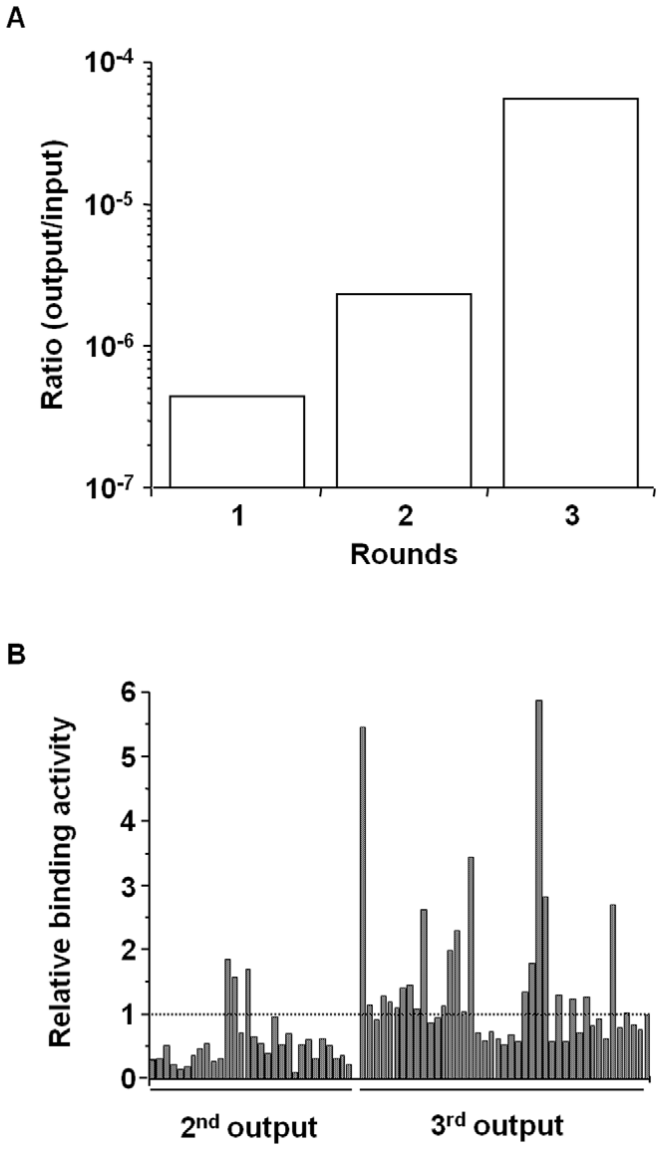
Screening of a novel CL4 binder. A) Enrichment of phages with affinity to CL4-BV. CL4-BVs coated on immunotubes were incubated with the C-CPE-derivative phage library at 1.6×10^12^ CFU titer (1^st^ input phage). The phages bound to CL4-BV were recovered (1^st^ output phage). The CL4-BV-binding phages were subjected to two additional cycles of the incubation and wash step, resulting in 2^nd^, 3^rd^ output phage. The ratio of output phage to input phage titers was calculated. B) Monoclonal analysis of C-CPE-derivative phage. CL4-BV-bound phage clones were isolated from the 2^nd^ and 3^rd^ output phages, and the interaction of the monoclonal phage with CL4-BV was examined by ELISA with anti-M13 antibody as described in Materials and methods. Data are expressed as relative binding to that of C-CPE-phage indicated by the most right column.

Then, we screened the CL4-binding phage by their affinity to CL4-BV. After addition of the C-CPE library to CL4-BV-adsorbed tubes, the CL4-BV-bound phages were recovered (1^st^ screening). We repeated this screening process two more times (2^nd^ screening and 3^rd^ screening). If the number of CL4-bound phage is increased during the screening, the ratio of the incubated phage titers to the recovered phage titers will increase. As shown in Fig. 3A, the ratio was increased during screening from 4.5×10^−7^ to 5.5×10^−5^, indicating that the screening system for CL4 binders may work. Indeed, the number of monoclonal phage clones with high affinity to CL4-BV was increased after the 3^rd^ screening compared with that after the 2^nd^ screening (Fig. 3B).

**Figure 4:**
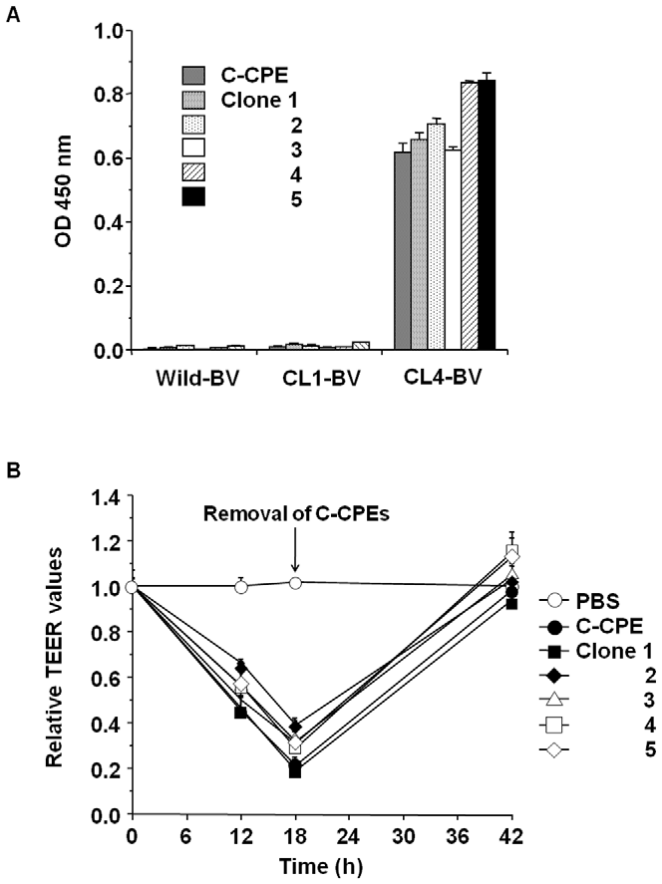
Isolation of a novel CL4 modulator. A) Interaction of the C-CPE derivatives with CL4. C-CPE derivatives were prepared as his-tagged recombinant proteins. The C-CPE derivatives (0.02 µg) were added to CL-BV-coated immunoplates, followed by detection of the C-CPE derivatives bound to CL-BV. Data are means ± SD (n = 4). B) Modulation of tight junction-barriers. Caco-2 cells were cultured on Transwell™ chambers. When TEER values reach a plateau, the cells were treated with C-CPE or C-CPE derivatives at the indicated concentrations. After 18 h of exposure to the C-CPEs, the cells were washed with medium to remove C-CPEs, and then the cells were cultured for an additional 24 h. Changes in TEER values were monitored during the C-CPEs treatment. Relative TEER values were calculated as the ratio of TEER values at 0 h. Data are representative of two independent experiments. The data are means ± SD (n = 4).

We analyzed the sequences of the CL4-BV-bound phages and got novel CL4-binder candidates with amino acid sequences that differed from the wild-type sequence (Table 2). To investigate their CL4-binding, we prepared the recombinant proteins of the binders and investigated their interaction with CL4 by ELISA with CL-BVs. As shown in Fig. 4A, the novel C-CPE derivatives had affinity to CL4 but not CL1. Next, we investigated whether the novel CL4 binders modulate TJ barrier in Caco-2 monolayer cell sheets, a popular model for the evaluation of TJ barriers [25]. Treatment of the cells with C-CPE resulted in decreased transepithelial electrical resistance (TEER) values, a marker of TJ integrity, and the TEER values increased after removal of C-CPE. The C-CPE derivatives (clones 1–5) had TJ-modulating activity similar to that of C-CPE (Fig. 4B).

**Table 2:** CL4-binding phages.

	304	305	307	309	313	318
C-CPE	S	S	S	N	S	K
Clone 1	R	V	S	A	R	R
2	R	S	V	A	R	K
3	G	D	G	R	T	R
4	S	A	P	R	S	A
5	R	S	L	K	S	K

The sequences of C-CPE mutant in the CL4-binding phages were analyzed.

## Discussion

CL is a promising target for pharmaceutical therapy. However, CL has low antigenicity, and there has been little success in the preparation of monoclonal antibody against the extracellular loop region of CL. The three-dimensional structure of CL has never been determined, so it is impossible to perform a theoretical design of a CL binder based on the structural information. In the present study, we developed a novel screening system for CL binders by using a BV system and a C-CPE phage display library, and we used this system to identify novel CL4 binders.

In ligand screening, the preparation of a receptor for the ligand is very critical. Membrane proteins are especially difficult to prepare as recombinant protein with an intact structure. Functional membrane proteins such as cell-surface proteins are heterologously expressed on BV in their native forms [19]–[21]. Interactions between membrane proteins can be detected by using receptor-displaying and ligand-displaying BV [21]. In the present report, we found that CL4-BV interacts with a CL4 binder, C-CPE, but it does not interact with C-CPE303 that lacks the CL4-binding residues of C-CPE. The CL4-binding site of C-CPE corresponds to that of CPE; so, the second extracellular loop of CL appears to be the C-CPE-binding site [23], [26]. These findings indicate that CL4 displayed on BV may have native form. We anticipate that CL-BV will be useful for the preparation of CL binders, such as peptides and antibodies.

To the best of our knowledge, the preparation of CL binder has been performed by only four groups. Offner et al. prepared polyclonal antibodies against extracellular domains of CL3 and CL4 [27], Ling et al. screened peptide types of CL4 binder by using a 12-mer peptide phage display library and CL4-expressing cells [28], Suzuki et al. generated a monoclonal antibody against the second extracellular loop of CL4 from mice immunized with a human pancreatic cancer cell line [29] and Romani et al. screened scFv against CL3 by using a human antibody phage display library [30]. However, the CL modulators have never been developed; thus, C-CPE is the only known CL4 modulator [12]. In the present study, we prepared a C-CPE phage library containing C-CPE mutants in which each of the 6 functional amino acids was randomly replaced with an amino acid, and we isolated CL4 binders by using CL4-BV as a screening ligand. Interestingly, all of the CL4 binders modulated TJ barriers. We are investigating why the substitution of the amino acids with the other amino acids modulated CL4. These findings indicate that a BV screening system with a C-CPE library may be a powerful method to develop CL modulators.

The CL family forms various types of TJ barriers through combinations of its more than 20 members in homophilic/heterophilic CL strands [31], . Intercellular proteins ZO-1 and ZO-2 determine the localization of CL strands [33]. If a screening system to reconstitute heterogeneous CL strands with ZO-1 and/or ZO-2 is developed, then useful and effective CL modulators can be identified. In this point, the BV system has extremely superior features. G protein and G protein-coupled receptors have been functionally reconstituted in BV [20], [34], and functional γ-secretase complexes have also been reconstituted on BV [35]. In the near future, the reconstituted CL system on BV will be developed and used for the screening of CL binders and modulators, hopefully leading to breakthroughs in pharmaceutical therapies that target CLs.

## Materials and Methods

### Recombinant BV construction and Sf9 cell culture

Recombinant BV was prepared by using the Bac-to-Bac expression system, according to the manufacturer's instructions (Invitrogen, Gaithersburg, MD). Briefly, mouse CL1 and CL4 cDNA (kind gifts from Dr. M Furuse, Kobe University, Japan) were inserted into pFastBac1, and the resulting plasmids were transduced into DH10Bac *E. Coli* cells. Recombinant bacmid DNA was extracted from the cells. Sf9 cells were transduced with the bacmid coding CL, and the recombinant BV was recovered by centrifugation of the conditioned medium [36].

### Preparation of the BV fractions

Sf9 cells (2×10^6^ cells) were infected with recombinant BV at a multiplicity of infection of 5. Seventy-two hours after infection, the BV fraction was recovered from the culture supernatant of infected Sf9 cells by centrifugation. The pellets of the BV fraction were resuspended in Tris-buffered saline (TBS) containing 1% protease inhibitor cocktail (Sigma-Aldrich, St. Louis, MO) and then stored at 4°C until use. The expression of CL1 and CL4 in the BV was confirmed by sodium dodecyl sulfate-polyacrylamide gel electrophoresis (SDS-PAGE) and immunoblot analysis with anti-CL antibodies (Zymed Laboratory, South San Francisco, CA).

### Preparation of mutant C-CPE library

C-CPE fragments in which the functional amino acids (S304, S305, S307, N309, S313 and K318) [24] were randomly mutated were prepared by polymerase chain reaction (PCR) with pET-H_10_PER as a template, a forward primer (5′-catgccatggccgatatagaaaaagaaatccttgatttagctgctg-3′, Nco I site is underlined) and a reverse primer (5′-ttttccttttgcggccgcaaa*snn*ttgaaataatat*snn*ataagggta*snn*tcc*snn*ata*snnsnn*attagcttt-3′, Not I site is underlined, and the randomly mutated amino acids are in italics). The PCR fragments were inserted into a pY03 phagemid at the NcoI/NotI sites [22]. The resultant phagemid containing the C-CPE mutant library was transduced into *E. coli* TG1 cells, and then the cells were stored at −80°C.

### Preparation of phage

TG1 cells containing phagemid coding a scFv, C-CPE, C-CPE mutant or C-CPE mutant library were culture in 2YT medium containing 2% glucose and ampicillin. When the cells grew to a growing phage, M13K07 helper phages (Invitrogen) were added, and the medium was changed into 2YT medium containing ampicillin and kanamycin. After an additional 6 h of culture, the phages in the conditioned medium were precipitated with polyethylene glycol. The phages were suspended in phosphate-buffered saline (PBS) and stored at 4°C until use.

### ELISA

Wild-BVs or CL-BVs (0.5 µg/well) were adsorbed onto an immunoplate (Greiner Bio-One, Frickenhausen, Germany). The wells were washed with PBS and blocked with TBS containing 1.6% BlockAce (Dainippon Sumitomo Pharma, Osaka, Japan). C-CPEs or phages were incubated in the immunoplate, and the BV-bound C-CPEs or phages were detected by using anti-his-tag antibody (Novagen, Darmstadt, Germany) or anti-M13 antibody (Amersham-Pharmacia Biotech, Uppsala, Sweden), respectively, horseradish peroxidase-labelled secondary antibody and TMB peroxidase substrate (Nacalai Tesque, Kyoto, Japan). The immunoreactive C-CPEs or phages were quantified by the measurement of absorbance at 450 nm. In the screening of phages, the data were normalized by the amounts of phages, which were quantified by ELISA for the FLAG-tag contained in the coat protein.

### Selection of phage by using BV

A total of 0.5 µg of BV was adsorbed onto an immunotube (Nunc, Roskilde, Denmark). The tube was washed with PBS and blocked with TBS containing 4.0% BlockAce. The BV-coated tubes were incubated with mixture of phages, and then the tubes were washed 15 times with PBS and 15 times with PBS containing 0.05% Tween 20. The phages bound to the tube were eluted with 100 mM HCl. TG1 cells were infected with the eluted phages, and phages were prepared as described above. The resulting phages were subjected to repeated selection by using the BV-coated immunotubes.

### Identification of a phage clone

To identify an isolated phage clone, we performed PCR or sequencing analysis. We amplified the inserted fragment into the phagemid by PCR using forward primer 5′-caggaaacagctatgac-3′ and reverse primer 5′-gtaaatgaattttctgtatgagg-3′. The resultant PCR products were subjected to agarose gel electrophoresis followed by staining with ethidium bromide. We performed a sequence analysis with primer 5′-gtaaatgaattttctgtatgagg-3′.

### Measurement of phage titer

To quantify the concentration of phages, we measured the titer (colony formation unit (CFU)/ml) of the phage solution. Briefly, the phage solution was diluted to 10^−5^–10^−10^ with PBS. The diluted solution was seeded onto Petrifilm™ (Tech-Jam, Osaka, Japan). After 24 h of incubation, the colonies were counted, and the titer was calculated.

### Purification of C-CPE mutants

C-CPE and C-CPE303, in which the CL-4 binding region of C-CPE was deleted, were prepared as described previously [13]. To prepare plasmid containing C-CPE mutants, the C-CPE mutant fragment was PCR-amplified by using phagemids coding C-CPE mutants as a template. The resulting PCR fragment was inserted into pET16b, and the sequence was confirmed. The plasmids were transduced into *E. coli* strain BL21 (DE3), and production of mutant C-CPEs was induced by the addition of isopropyl-D-thiogalactopyranoside. The harvested cells were lysed in buffer A (10 mM Tris-HCl, pH 8.0, 400 mM NaCl, 5 mM MgCl_2_, 0.1 mM phenylmethanesulfonyl fluoride, 1 mM 2-mercaptoethanol, and 10% glycerol) that was supplemented with 8 M urea when necessary. The lysates were applied to HiTrap™ Chelating HP (GE Healthcare, Buckinghamshire, UK), and mutant C-CPEs were eluted with buffer A containing 100–400 mM imidazole. The buffer was exchanged with PBS by using a PD-10 column (GE Healthcare), and the purified protein was stored at −80°C until use. Purification of the mutant C-CPEs was confirmed by SDS-PAGE, followed by staining with Coomassie Brilliant Blue and by immunoblotting with anti-his-tag antibody (Novagen). Protein was quantified by using a BCA protein assay kit with bovine serum albumin as a standard (Pierce Chemical, Rockford, IL).

### TEER assay

Caco-2 cells were seeded in Transwell™ chambers (Corning, NY) at a subconfluent density. The TEER of the Caco-2 monolayer cell sheets on the chamber was monitored by using a Millicell-ERS epithelial volt-ohmmeter (Millipore, Billerica, MA). When TEER values reached a plateau, indicating that TJs were well-developed in the cell sheets, the Caco-2 monolayers were treated with C-CPE or C-CPE mutants on the basal side of the chamber. Changes in TEER values were monitored. The TEER values were normalized by the area of the Caco-2 monolayer, and the TEER value of a blank Transwell™ chamber (background) was subtracted.
